# Quantitative Method for Monitoring Tumor Evolution During and After Therapy

**DOI:** 10.3390/jpm15070275

**Published:** 2025-06-28

**Authors:** Paolo Castorina, Filippo Castiglione, Gianluca Ferini, Stefano Forte, Emanuele Martorana

**Affiliations:** 1 Istituto Nazionale Fisica Nucleare, Sezione di Catania, 95123 Catania, Italy; 2 Faculty of Mathematics and Physics, Charles University, V Holešovičkách 2, 18000 Prague 8, Czech Republic; 3 Istituto Oncologico del Mediterraneo, 95128 Viagrande, Italy; stefano.forte@grupposamed.com (S.F.); emanuele@martorana.email (E.M.); 4 Biotechnology Research Center, Technology Innovation Institute, Yas Island, Abu Dhabi P.O. Box 9639, United Arab Emirates; filippo.castiglione@tii.ae; 5 Institute for Applied Computing, National Research Council of Italy, Via dei Taurini 19, 00185 Rome, Italy; 6 REM Radioterapia, 95029 Viagrande, Italy; gianluca.ferni@grupposamed.com; 7 Department of Medicine and Surgery, University of Enna Kore, 94100 Enna, Italy

**Keywords:** tumor growth, monitoring treatment response, predictive personalized tumor progression, support clinical decision-making

## Abstract

**Objectives**: The quantitative analysis of tumor progression—monitored during and immediately after therapeutic interventions—can yield valuable insights into both long-term disease dynamics and treatment efficacy. Methods: We used a computational approach designed to support clinical decision-making, with a focus on personalized patient care, based on modeling therapy effects using effective parameters of the Gompertz law. **Results**: The method is applied to data from in vivo models undergoing neoadjuvant chemoradiotherapy, as well as conventional and FLASH radiation treatments. **Conclusions**: This user-friendly, phenomenological model captures distinct phases of treatment response and identifies a critical dose threshold distinguishing complete response from partial response or tumor regrowth. These findings lay the groundwork for real-time quantitative monitoring of disease progression during therapy and contribute to a more tailored and predictive clinical strategy.

## 1. Introduction

Cancer remains one of the leading causes of mortality worldwide, accounting for approximately 10 million deaths in 2022, according to the World Health Organization [1]. In Europe, cancer represents a major public health concern, with over 4 million new cases and nearly 2 million cancer-related deaths recorded annually [2]. The most commonly diagnosed cancers in Europe include breast, colorectal, prostate, and lung cancers, which together account for more than half of the cancer burden across the continent. Notably, colorectal cancer is the second leading cause of cancer death in Europe, with marked disparities in incidence and survival rates between Western and Eastern European countries, reflecting differences in screening programs, healthcare infrastructure, and access to innovative treatments. Such regional statistics provide critical context and underscore the pressing need for improved diagnostic and therapeutic strategies. This reinforces the significance of ongoing research aimed at integrating precision oncology into clinical practice, with the goal of improving patient outcomes across diverse European populations.

Cancer is a complex disease impacted by various factors, including genetic, environmental, and lifestyle determinants. At a biological level, it is affected by tumor heterogeneity, micro-environmental features, and adaptive evolution. These complexities make both clinical decisions and the development of procedures and treatments more difficult. To overcome this challenge, significant efforts have been made in recent decades to establish a patient-oriented approach, with a strong focus on precision medicine, driven by advancements in molecular profiling, targeted therapies, and bioinformatics algorithms. The combination of biology, computer science, mathematics, and medicine plays a crucial role in developing computational tools to support clinical decisions and therapeutic indications [3,4,5,6,7,8,9,10].

Specifically, in silico models are widely applied to predict tumor growth and therapy effects [11]. However, to be effective, they need to meet the following criteria: (a) reliability, meaning that they should produce consistent and accurate predictions; (b) sufficient validation, that is, they should be tested and confirmed against experimental and/or clinical data; (c) applicability to different phenotypes, meaning that they should work for various types of tumors and account for individual patient differences; (d) easy to use, or user-friendly for clinicians.

Although clinical predictions are typically associated with biomarkers, recent publications highlight the importance of accurately analyzing initial tumor evolution during therapy, with the aim of using simple algorithms to make long-term predictions [5,12,13,14,15].

In fact, improved diagnostic techniques can provide comprehensive data on tumor evolution, enabling the application of computational models to discern patterns and correlations, thus predicting treatment responses and future disease development more accurately. Notable examples include T2-weighted and diffusion-weighted (DWI) magnetic resonance imaging (MRI) [5,13,16].

Currently, monitoring of all therapies, including surgery, radiation therapy, chemotherapy and immunotherapy, appears to rely on statistically-determined schedules. For example, the waiting time for colorectal cancer surgery was originally estimated at 8 weeks, but a delay of approximately 12 weeks is now considered clinically more appropriate [17]. Similarly, in immunotherapy, evolution assessments occur every three months, a standardized procedure that rarely meets the therapeutic needs of everyone [18,19].

However, initiating a comprehensive patient monitoring protocol at the beginning of treatment allows for real-time therapeutic adjustments in response to tumor evolution and patient outcomes. This proactive approach, often overlooked, has the potential to significantly enhance clinical outcomes and quality of care by prioritizing the individual needs and responses of each patient. In fact, quantitative monitoring during therapy is rarely used as a predictive tool primarily because most mathematical models in the literature [20,21,22,23] rely on systems of differential equations (ordinary, partial or stochastic) that depend on a large number of parameters and initial conditions (typically greater than ten), making them challenging to apply in clinical personalized decision-making. Likewise, computational simulations and agent-based models often involve a complex set of parameters.

Another important area that warrants closer attention is the quantitative assessment of therapeutic effects in radiotherapy. For example, the development of radiation techniques (i.e., 3D conformal radiotherapy, 3D-CRT, intensity-modulated radiotherapy, IMRT, volumetric arc radiotherapy, VMAT, stereotactic body radiotherapy, SBRT [24,25,26]) able to perform precision radiation delivery, aiming to maximally spare organs at risk (OARs) close to the target volume [27], has allowed for the escalation of the radiation dose in some anatomical sites, resulting in better tumor control and decreased toxicity [28]. As tight as the set-up margins can be around the target volume so as not to miss it in precision radiation delivery techniques, it is still unavoidable that some high-risk doses will be deposited in healthy tissue and organs closely abutting the tumor. Therefore, a quantitative analysis of the therapy effect is a relevant complementary tool [29,30,31].

In this paper, we suggest that tracking the progression of the disease through quantitative monitoring during and immediately after the end of the therapy, with a limited number of parameters and a straightforward fitting process, can offer important insights into how the disease is evolving and how effective the treatment is. This approach has the potential to lead to a valuable and user-friendly tool for clinical decision-making, as it offers a more comprehensive understanding of the long-term effects of therapy.

More precisely, the computational method—whose mathematical formulation is presented in the following section and in the Supplementary Material—is based on a macroscopic tumor growth model governed by the Gompertz law. This model relies on two effective parameters that capture the influence of therapy: the change in the GL parameters relative to the growth in the control group which reflects the impact of the therapy, during the course of the treatment. By deriving their values from early treatment-response data, the approach enables long-term predictions of disease progression. Estimating these quantities does not require specialized software; any fitting routine based on the proposed equations can be employed. Compared to microscopic models, the key advantage is the simplicity afforded by the limited number of parameters, though this comes at the cost of a less detailed depiction of biodynamic mechanisms. In essence, while biologically microscopic models offer greater explanatory power regarding underlying processes, they often do so at the expense of clinical applicability at the personalized level.

The method of effective growth parameters is well-known in population dynamics [32,33,34,35] and applied in epidemic spreading models [36] and oncology (see for example [12,15,37,38]).

Our analysis begins by modeling the growth of cancer stem cells (CSCs), derived from lung cancer tissue samples, after implantation in a mouse model and exposure to varying doses and dose rates of conventional radiotherapy [39]. We then quantitatively investigate the impact of FLASH radiotherapy using a macroscopic approach, identifying distinct stages of treatment response and determining a critical dose threshold for complete response (CR) and partial response (PR).

The proposed method is subsequently applied to neoadjuvant radiochemotherapy. For completeness, we first revisit predictions of the late-stage behavior of colorectal cancer following radiochemotherapy, as presented in [5,12], demonstrating how a quantitative macroscopic analysis of tumor progression during and shortly after therapy can support surgical decision-making.

Finally, the application of the approach to dose–response analysis in renal carcinoma aims to demonstrate how it can be applied in a patient-centered manner, including specific examples and discussing the accuracy of long-term predictions.

## 2. Materials and Methods

### Computational Setting

The proposed phenomenological method is based on the mathematical formulation reported in detail in the Supplementary Materials S1 and S2.

An untreated tumor volume, *V* grows in time *t* according to the Gompertz law (GL) [40,41,42,43]

(1) $$V\left(t\right)=V\left(t_{0}\right)e^{\left[ln\frac{V_{\infty }}{V(t_{0})}\right]\left[1-e^{-k(t-t_{0})}\right]},$$

The solution of the Gompertz Equation (S1), S1, depends on the two parameters $V_{\infty }$, the carrying capacity (CC), and *k*, related to the reduction of the initial exponential growth rate.

The choice of the GL is irrelevant to the general discussion since any other sigmoid-like curve with two parameters, such as the logistic one, can be applied. The GL gives a better fit to macroscopic cancer growth [42].

If we account for the effect of the therapy, F(t), the cancer growth formula becomes (see Equations (S2)–(S4)) S1

(2) $$V\left(t\right)=V\left(t_{0}\right)e^{\left[ln\frac{V_{\infty }}{V(t_{0})}\right]\left[1-e^{-k(t-t_{0})}\right]-\int_{t_{0}}^{t}dt^{\prime }F\left(t^{\prime }\right)e^{-k(t-t^{\prime })}}.$$

The limit t→∞ mathematically defines the complete response case (CR) and the partial response case (PR). Indeed, one obtains CR or PR if

(3) $$ln\frac{V_{\infty }}{V(t_{0})}-lim_{t\to \infty }\int_{t_{0}}^{t}dt^{\prime }F\left(t^{\prime }\right)e^{-k(t-t^{\prime })}$$

goes to −∞ or to a negative finite value, respectively. For example, if F(t)≃t, one obtains CR [44,45], while for constant $F\left(t\right)=F_{0}$ one obtains PR when $ln(V_{\infty }/V\left(t_{0}\right)-F_{0}/k<0$. The limit of time going to infinity is, obviously, purely formal. What is important is the long-term effects of the therapy and F(t)≃t is a mathematical example to show that a cumulated therapy effect which increases linearly in time gives a complete recovery. Indeed, this F(t) behavior corresponds to the Norton–Simon [44] hypothesis, to obtain an exponentially decreasing tumor volume.

The crucial aspect of the proposed computational method is that the effects of therapy (i.e., the contribution of the function F(t) in Equation (2)), which summarizes many biological effects, can also be incorporated into the definition of the GL effective parameters, according to the following evolution pattern:

(4) $$V\left(t\right)=V\left(t_{0}\right)e^{\left[ln\frac{V_{\infty }^{eff}}{V(t_{0})}\right]\left[1-e^{-k_{eff}\left(t-t_{0}\right)}\right]}.$$

where $V_{\infty }^{eff}$ and $k_{eff}$ are related to the cumulative effect of the therapy (i.e., the time integral of F(t) in Equation (2)).

The relationship between the effective parameters and the effects of the therapy, i.e., between Equations (2) and (4), is discussed in detail in Supplementary Material S1 and S2, in a completely general mathematical formulation, including the time dependent case. Alternatively, the data can be more precisely fitted using Equation (2) or Equation (4), depending on the size of the data set, its temporal characteristics, and the number of free parameters (see below).

In general, monitoring conditions and potential evolution in clinical therapy protocols requires further methodological analysis to properly evaluate the effects of the therapy.

Indeed, regardless of the specific fractionization, a therapy is administrated at regular intervals, Δt at times $t_{1},t_{2},\ldots ,t_{n}$. Let us call $V\left(n^{-}\right),V\left(n^{+}\right)$ the tumor volume before and after the *n*-th treatment.

According to the outlined methods, one needs to know at the beginning the GL parameters of the control group to accurately determine the effects of the therapy. Therefore, after the measurement of the tumor size at the diagnosis, V(0), one has to observe the volume at $\tau_{1}$, $V(\tau_{1})$ ($0<\tau_{1}<t_{1}$), and before the first treatment $V(1^{-})$ at time $t_{1}$.

At $t_{1}$ the the first dose is administrated and accordingly to Equation (2), the tumor volume before the second dose, $V(2^{-})$, is given by

(5) $$V\left(2^{-}\right)=V\left(1^{-}\right)e^{\left[ln\frac{V_{\infty }}{V(1^{-})}\right]\left[1-e^{-k\Delta t}\right]-RT\left(1\right)}$$

where RT(1) is the effect of the first dose in the time interval $t_{2}-t_{1}$, i.e.,

(6) $$RT\left(1\right)=R\left(t_{1},t_{2}\right)=\int_{t_{1}}^{t_{2}}dt^{\prime }F\left(t^{\prime }\right)e^{-k(t_{2}-t^{\prime })}.$$

RT(1) can be estimated by a measure of $V(2^{-})$, since $V\left(1^{-}\right),V_{\infty },k$ have been previously evaluated. After the second dose at time $t_{2}$, the evolution before the third dose gives

(7) $$V\left(3^{-}\right)=V\left(2^{-}\right)e^{\left[ln\frac{V_{\infty }}{V(2^{-})}\right]\left[1-e^{-k\Delta t}\right]-RT\left(2\right)}$$

where RT(2) is the effect of the second dose in the time interval $t_{3}-t_{2}$. Again, RT(2) can be estimated by a $V(3^{-})$ measure.

Let us clarify that the volume $V(3^{-})$ contains the cumulative effect of the therapy in the interval $\Delta t=t_{2}-t_{1}$ and $\Delta t=t_{3}-t_{2}$. Indeed, by substitution of Equation (5) in Equation (7) one obtains

(8) $$V\left(3^{-}\right)=V\left(1^{-}\right)e^{\left[ln\frac{V_{\infty }}{V(1^{-})}\right]\left[1-e^{-k(t_{3}-t_{1})}\right]-RT\left(1\right)e^{-k(t_{3}-t_{2})}-RT\left(2\right)}$$

where $t_{3}-t_{1}=2\Delta t$ and $t_{3}-t_{2}=\Delta t$ for fixed time interval between doses. The first term in the exponential is the no-treatment growth. Therefore the cumulative effect in the second time interval $t_{3}-t_{2}$ contains a reduction of the therapy effect during the first interval $t_{2}-t_{1}$, RT(1).

The size evolution $V\left(2^{-}\right)/V\left(1^{-}\right),V\left(3^{-}\right)/V\left(2^{-}\right),\ldots$ depends on two contributions; see Equation (S27) Supplementary Material S2: the regrowth, RG, given in general by

(9) $$RG=exp\left\{\left(ln\frac{V_{\infty }}{V[{\left(n-1\right)}^{-}]}\right)\left[1-e^{-k\Delta t}\right]\right\}$$

and the therapy effects

(10) $$TH=exp\left\{RT(t_{n},t_{n-1})\right\}.$$

Notice also the change in the condition of the tumor size reduction during therapy with respect to the initial size $V(1^{-})$. By imposing $V\left(n^{-}\right)<V\left(1^{-}\right)$, n=2,3, …, the condition before the second dose is

(11) $$\left[ln\frac{V_{\infty }}{V(1^{-})}\right]\left[1-e^{-k\Delta t}\right]<RT\left(1\right)$$

and before the third dose, according to Equation (8), it is given by

(12) $$\left[ln\frac{V_{\infty }}{V(1^{-})}\right]\left[1-e^{-2k\Delta t}\right]<RT\left(1\right)e^{-k\Delta t}+RT\left(2\right).$$

The procedure is iterative (see Supplementary Material) and the estimate of the series RT(1),RT(2),…,RT(n) gives the cumulative effect after *n* doses, $RT_{tot}\left(n\right)$:

(13) $$V\left(n^{-}\right)=V\left(1^{-}\right)e^{\left[ln\frac{V_{\infty }}{V(1^{-})}\right]\left(1-e^{-(n-1)k\Delta t}\right)-RT_{tot}\left(n\right)}$$

where

(14) $$RT_{tot}\left(n\right)=\Sigma_{m=1}^{n-1}RT\left(m\right)exp\left[-k\Delta t\left(n-\left(m+1\right)\right)\right]$$

and the condition for tumor regression, in general, is

(15) $$ln\frac{V_{\infty }}{V(1^{-})}\left[1-e^{-(n-1)k\Delta t}\right]<RT_{tot}\left(n\right).$$

If, on the other hand, one requires a continuous size reduction, i.e., $V\left[n^{-}\right]<V\left[{\left(n-1\right)}^{-}\right]<V\left[{\left(n-2\right)}^{-}\right]<\ldots V\left[1^{-}\right]$, the previous condition becomes Equation (S32) in Supplementary Material S2.

Monitoring the volume evolution during therapy can provide clinical insights into CR, PR or regrowth.

In what follows, we show how the proposed method supports clinical decision-making recalling that its ability to describe the dynamics of macroscopic changes in human tumors was assessed using time-series data available in the scientific literature. Due to the rarity of tumor growth data sets that include accurate longitudinal volumetric measurements, which are not routinely collected, only a limited number of in vitro and in vivo studies were identified. These studies provided data for in vivo/human tumors treated with either conventional radiotherapy [39], FLASH radiotherapy [46], radiochemotherapy [5] or chemotherapy [47] and included measurements for lung, colorectal, and renal tumors. The parameters are determined by minimizing the χ-squared value using the Grace 5.1.22 software for Linux, although any other routine may be employed as well, and their errors refer to a standard deviation.

## 3. Results

### 3.1. Analysis of In Vivo Conventional Radiotherapy

The growth data in our previous work [39] pertain to both in vitro and in vivo experiments involving CSCs isolated from lung cancer. Here, we use in vivo data from mouse models obtained by transplantation of human CSCs. These data include 19 mice, each bearing a human tumor derived from CSC implantation. The animals were distributed as follows: eight per treatment group and three in the control group. They were subjected to radiation treatment, with the dose and rate selected based on those that demonstrated sensitivity in vitro, using the lowest effective dose (LED) and the highest ineffective dose (HID).

In detail, implanted mice were exposed to radiation from 5 to 10 Gy with a single rate of 2400 MU/min (radiation dose (MU) delivered per unit time (min); 100 MU are equivalent to 1 Gy of absorbed dose) and tumor growth was measured twice a week, by an external digital caliper.

The in vivo evolution of untreated tumor cell lines is analyzed at first. The doses are then gradually increased, starting from the ineffective dose (HID) until a response is observed (i.e., the LED). As the radiation dose increases, the response of the tumor is carefully monitored, and at a certain dose level, a noticeable tumor regression is observed.

We identify a threshold or critical dose, such that the tumor evolves towards complete response, which varies depending on the type of tumor, its location, and individual factors.

For this small data set (three experimental points for single cell line, with increasing or decreasing monotonic behavior [39]), we apply the approach of the effective Gompertz law (GL) parameters (see Section 2) by fitting according to Equation (4) (the lower index denotes the dose, *d*), i.e.,

(16) $$V_{d}\left(t\right)/V\left(0\right)=e^{\left[ln\left(V_{d}^{\infty }/V\left(0\right)\right)\right]\left[1-exp\left(-k_{d}t\right)\right]}.$$

where $V_{d}$ is the volume. $V_{d}^{\infty },k_{d}$ are the effective dose-dependent parameters. For positive $k_{d}$, the critical dose is given by the condition $ln(V_{d}^{\infty }/V\left(0\right))<0$, i.e., $V_{d}^{\infty }<V\left(0\right)$. On the other hand, if $k_{d}t\ll 1$, the GL reduces to an exponential behavior, since the previous equation gives

(17) $$V_{d}\left(t\right)/V\left(0\right)≃e^{[ln\left(V_{d}^{\infty }/V\left(0\right)\right]k_{d}t}.$$

In this respect, if the data sample is not large enough, it could be difficult to disentangle the two GL parameters and one can only determine the product $ln\left(V_{d}^{\infty }/V\left(0\right)\right)k_{d}$. The critical condition is therefore marked by a change in its sign.

**Figure 1:**
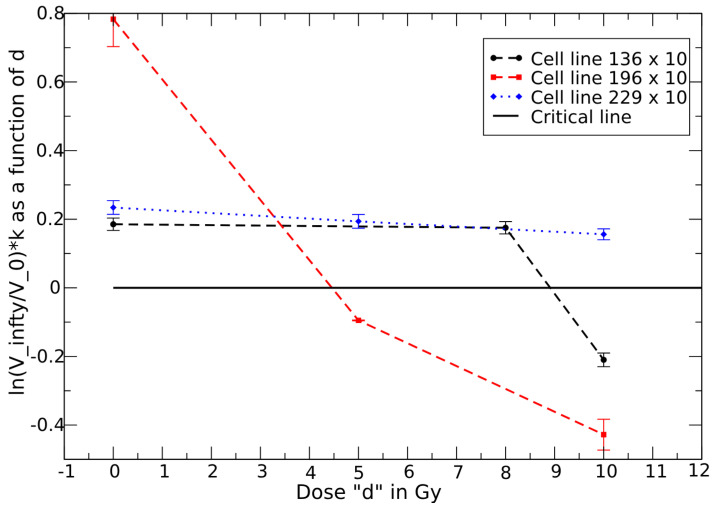
GL effective parameters as a function of dose *d* for cell lines 136,196,229 (multiplied by a factor 10). The crossing point with the critical line is the critical dose. The error bars represent the uncertainties in the parameters: Line 136: 0.0185±0.0015, 0.0175±0.0014, −0.0206±0.0017; Line 196: 0.0783±0.008, 0.0095±0.0005, 0.43±0.007; Line 226: 0.023±0.0024, 0.019±0.0018, 0.016±0.0015, in (day)^−1^, see Table 1.

**Figure 2:**
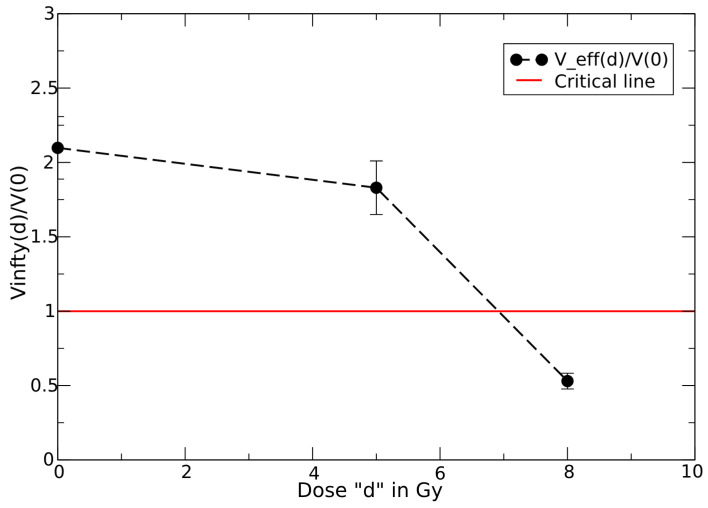
GL effective carrying capacity as a function of dose *d* for cell line 36. The crossing point with the critical line is the critical dose. The error bars represent the uncertainties in the parameter: Line 36: 2.1±0.22, 1.82±0.25, 0.53±0.04—see Table 1.

For each cell line and each single dose, one can easily fit the data in ref. [39] by the effective GL parameters, see Table 1 and Supplementary Material, and their dependence on *d*. The dose dependence can be described by the effective carrying capacity $V_{d}^{\infty }$ or by the combination $ln\left(V_{d}^{\infty }/V\left(0\right)\right)k_{d}$ given by

(18) $$V_{d}^{\infty }=V_{0}^{\infty }-cd^{\lambda },$$

or, for the exponential trend,

(19) $$ln\left(V_{d}^{\infty }/V\left(0\right)\right)k_{d}=ln\left(V_{0}^{\infty }/V\left(0\right)\right)k_{0}-cd^{\lambda },$$

where the constants c,λ and the resulting critical doses are reported in Table 1. The corresponding dose dependence is depicted in Figure 1 for cell lines 136,196,229 and in Figure 2 for cell line 36.

This example illustrates how a series of monitoring can aid in determining the critical dose.

### 3.2. Analysis of In Vivo FLASH Radiotherapy

FLASH therapy is an innovative form of radiotherapy whose improvement of radiation’s therapeutic ratio revolves around an exceptionally high dose rate [48,49,50,51]. It is a significant advance in tumor therapies, as it leads to a reduction in normal tissue toxicity, suggesting that a high-dose rate mimics cellular hypoxia that prevents damage in healthy tissues. In terms of effectiveness, compared to conventional radiotherapy (CONV-RT), FLASH-RT provides an equivalent anti-tumor result [52].

In particular, in [46], lung fibrogenesis in C57BL/6J mice exposed either to short pulses (≤500 ms) of radiation, delivered at ultrahigh dose rates (≥40 Gy/s, FLASH) or to conventional dose-rate irradiation (≤0.03 Gy/s, CONV) in single doses, have been investigated to determine the potential of ultrahigh dose-rate irradiation in radiotherapy.

**Figure 3:**
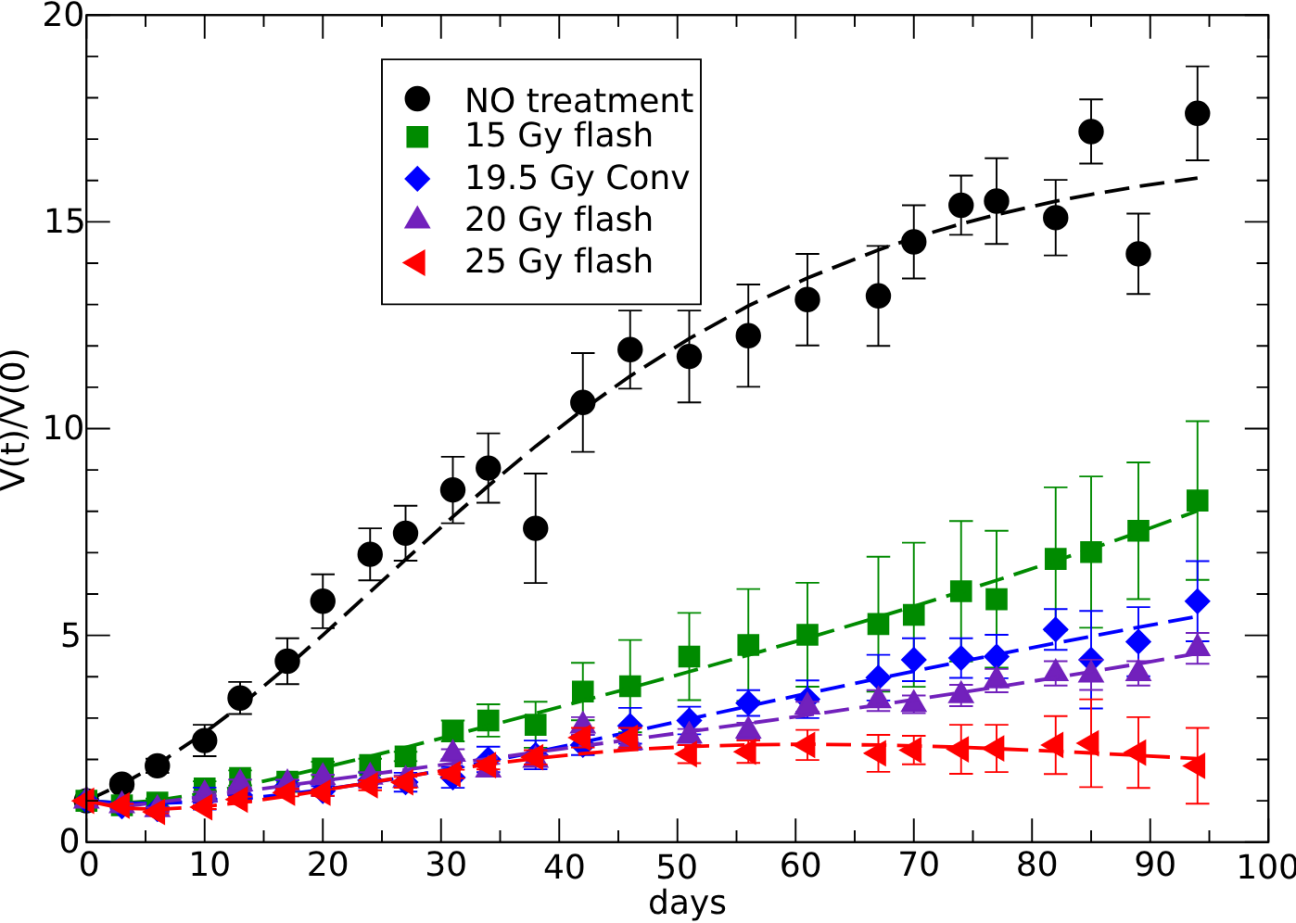
Fits (colored lines) of the FLASH and conventional therapy by the parameterization of F(t) in Equation (21). The data, from [46], give the ratio between the tumor volume and its initial value as a function of time after radiotherapy, including the experimental error (bars).

**Figure 4:**
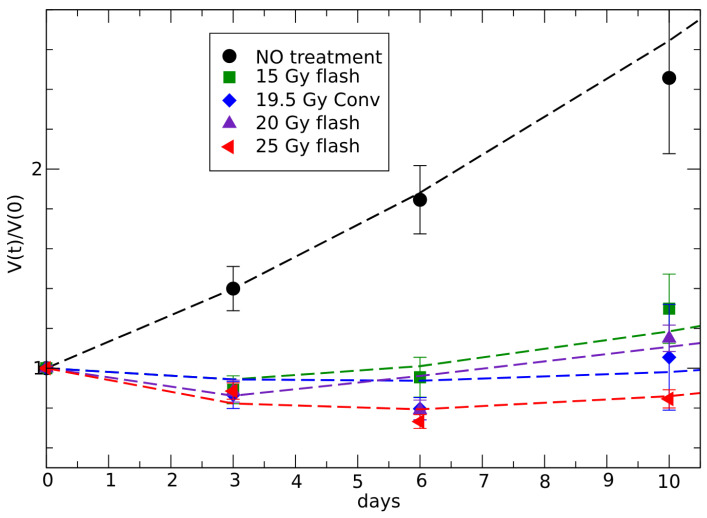
Radiotherapy effects in the first ten days of Figure 3. The days immediately following the dose primarily show the direct cell-killing effect of radiation, related with the parameters $c_{0}$ and $c_{1}$ in the phenomenological parametrization in Equation (20).

In vivo data [46] indicate different phases of the response to radiotherapy. Initially, there is a decrease in tumor volume due to the minimal impact of the specific regrowth rate. This is followed by a second phase where regrowth surpasses the reducing therapeutic effect. In the third phase, at later times, the response differentiates between complete response and partial response based on the radiation dose, see Figure 3 and Figure 4.

In accordance with the previous pattern and taking into account the lack of knowledge of all biological effects of this therapy, let us therefore apply the formulation in Equation (2) (see Section 2) with a phenomenological parameterization of the radiotherapy effect, suggested by the data trend, given by:

(20) $$F\left(t\right)=c_{0}+c_{1}\times e^{\left(-c_{2}t\right)}-c_{f}t$$

with parameters $c_{0},c_{1},c_{2},c_{f}$.

It is worth emphasizing that the parameterization mentioned above aligns with the definition of the effective parameters of GL, as illustrated in Supplementary Material S1. In practical terms, the fitting process is carried out using either Equation (2) or Equation (4), depending on the time behavior. For monotonic trends (consistently increasing or decreasing), it is more straightforward to apply Equation (4). However, if the evolution pattern is more complex, it is advisable to employ the fitting procedure with Equation (2), rather than deriving the effective parameters directly from the data, since their dependence on time can be difficult to identify. The relation between the cumulative effect (time integral of F(t)) and the GL effective parameters is given in Supplementary Material S1 and applied to the FLASH therapy in Supplementary Material S2.

To understand the role of $c_{0},c_{1},c_{2},c_{f}$, one has to substitute the previous formula in Equation (2) to obtain ($t_{0}=0$)

(21) $$V\left(t\right)/V\left(0\right)=exp[\left(ln\frac{V_{\infty }}{V(0)}-c_{0}/k-c_{f}/k^{2}\right)\left(1-e^{-kt}\right)-\frac{c_{1}}{k-c_{2}}\left(e^{-c_{2}t}-e^{-kt}\right)+tc_{f}/k].$$

The evolution immediately after the dose, for kt≪1 is given by

(22) $$V\left(t\right)/V\left(0\right)≃exp[\left(kln\frac{V_{\infty }}{V(0)}-c_{0}-c_{1}\right)t]$$

which describes the negative slope for $klnV_{\infty }/V\left(0\right)-c_{0}-c_{1}<0$ (notice that $c_{2}$ and $c_{f}$ play no role in this limit).

The parameter $c_{2}$ gives the decrease of the radiation effect at a larger, but intermediate, value of kt.

Finally, for kt≫1 one obtains

(23) $$V\left(t\right)/V\left(0\right)≃exp[\left(ln\frac{V_{\infty }}{V(0)}-c_{0}/k-c_{f}/k^{2}\right)+tc_{f}/k],$$

which shows the crucial role of the parameter $c_{f}$. Indeed, it is clear from Equations (21)–(23) that a negative $c_{f}$ increases the radiation effect leading to CR, $c_{f}=0$ describes PR, and a positive value produces its depletion. If $c_{f}$ is positive, regrowth begins once the exponent in Equation (23) turns positive. Depending on the relative contributions of the two terms in the exponential of Equation (23), the regrowth may occur long after therapy, resembling a partial recovery. However, with positive $c_{f}$, it will restart anyway and when (t=t*)

(24) $$c_{0}/k+c_{f}/k^{2}=t*c_{f}/k,$$

it reaches the same value of the untreated GL behavior and one assumes this trend for t>t*. Therefore $c_{f}$ is a crucial parameter to identify the critical dose.

The parameterization of F(t) should describe various effects of the therapy related to the underlying microbiological model. The phenomenological approach indicates that immediately after the dose, volume reduction is a direct effect of cell killing radiation, which decreases with time ($c_{0}$ and $c_{1}$). The intermediate regrowth implies that the repair system of the cellular damage overcomes the therapy. Late evolution (essentially the parameter $c_{f}$) should be associated, for example, with delayed radiotherapy effects or the immune response triggered by radiation. Indeed, decades of intensive research have led to the understanding that radiation therapy has the potential to interact with the immune system [53]. It is well-known that ionizing radiation produces double-strand DNA breaks. Once radio-induced DNA damage occurs, damage-associated molecular patterns are released. This results in an increase in inflammatory signaling, which triggers the activation of cells such as macrophages, which in turn stimulates the activation of cytotoxic T-cells, thereby promoting the killing of cancer cells.

The fit of data [46], according to Equation (21), is reported in Figure 3 and Figure 4, with the corresponding parameters in Table 2, giving the critical change of sign of $c_{f}$ in the region between 20 and 25 Gy FLASH doses.

The fit of the conventional therapy data could be performed by the effective GL curve, but for direct comparison with the FLASH therapy description it is better to apply the same parameterization to show that, indeed, there is only a small change in the effectiveness of the two therapies.

For FLASH therapy, the dose (in Gy) dependence of $c_{f}$ is (in day^−2^)

(25) $$c_{f}\left(d\right)=0.000515-2.17\times 10^{-7}{\left(d-15\right)}^{3.64}$$

which recalls a first-order phase transition, with the parameter $c_{f}$ as the order parameter. The critical dose (i.e., the change in sign of $c_{f}$) is 23.5 Gy.

### 3.3. Neoadjuvant Therapy in Colorectal Cancer

This section starts by recalling the role of monitoring the disease evolution in supporting patient-oriented, clinical, decisions by the analysis of the data in ref. [5] and, then, by applying the proposed approach to the data in ref. [54].

According to reference [5], eight patients with locally advanced rectal cancer underwent neoadjuvant chemoradiotherapy (CRT), resulting in either a complete pathological response (CR) or a partial response (PR). Daily evaluations of tumor volume were conducted using magnetic resonance imaging prior to the irradiation. The therapy effects immediately reduced the tumor volume, decreasing steadily during and after the treatment. The therapy surpasses the critical threshold, and the data were analyzed using GL effective parameters, as referenced in [14], providing clear evidence that the effective carrying capacity is much smaller for CR than for PR patients. This implies that in the volumetric analysis of tumor evolution, the rate of change (rather than the total volume) is a more reliable indicator of evolution. The important aspect is that reaching this conclusion does not require using all 28 observations. The analysis of approximately 50% of the data series can yield the same result in a patient-specific way [12]. In other words, a limited time series of tumor evolution during therapy can predict long-term evolution, providing valuable clinical insights.

Let us now apply the same method of effective GL parameters (see Section 2) to the data in ref. [54] where 15 patients with biopsy-proven adenocarcinoma of the rectum received standard preoperative CRT treatment with an irradiation dose of 50 Gy in 25 fractions of 2 Gy, delivered in 5 weeks on workdays combined with oral capecitabine, two times a day. Eight MRI scan sessions were performed for every patient to measure the pre-operative tumor volume regression: before therapy, weekly during 5 weeks of treatment, 2–4 weeks after CRT, and 7–8 weeks after CRT.

The data refer to the percentage of size reduction during and after therapy, up to approximately 90 days from the initial dose, and represent the average results across all patients. The analysis aims to demonstrate how the proposed computational approach can be applied. When patient-specific data are available, the method can be used to evaluate the disease evolution in a personalized manner.

**Figure 5:**
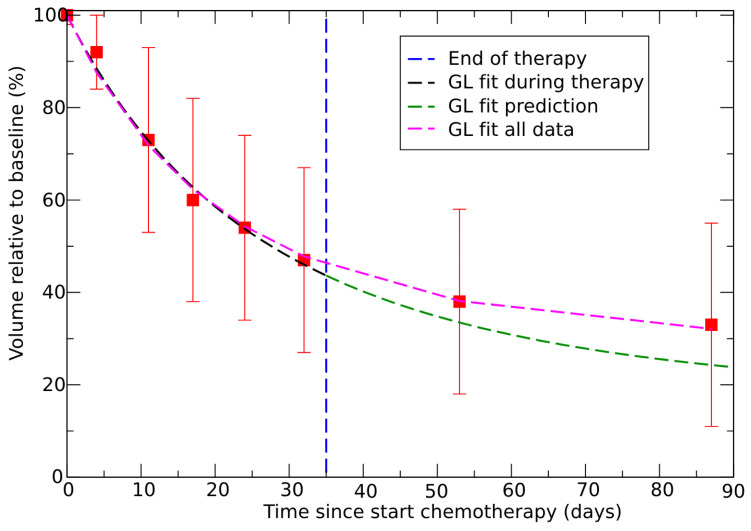
GL fit to the tumor volume regression for neoadjuvant therapy in colorectal cancer with the whole set of data (violet); GL fit of the tumor volume during therapy (black); prediction based on the parameters fitted during therapy (green). Data from ref. [54]. Effective parameters for the GL violet and green curves are $V_{\infty }=V_{0}exp\left(-1.243\right)$ and k=0.0282 and $V_{\infty }=V_{0}exp\left(-1.81\right)$ and k=0.0175 in day^−1^, respectively.

Initially, the entire data set was fitted with GL using effective parameters. A similar fit was performed, focusing only on the data during therapy. With the effective parameters obtained in this way, the outcome after the end of therapy was predicted. The results are depicted in Figure 5 and show good agreement between data and prediction, which implies the possibility of evaluating the outcome 55 days after the end of the therapy. A measurement taken immediately after the end of the therapy, for example, one week later, could further enhance the reliability of the prediction, as it can reveal any progression discontinuities caused by the cessation of the drug.

### 3.4. Dose–Response in Metastatic Renal Cell Carcinoma

Serial measurements of the sum of the longest tumor diameters (SLD), performed by Computed Tomography or MRI scans, from individual (RECORD-1 trial—NCT00410124) patients were performed in ref. [47] to establish the efficacy and safety of everolimus in metastatic renal cell carcinoma. The data support the conclusion that 10 mg daily is the preferred clinical dose of everolimus to reduce the SLD of target lesions.

Representative data of the measured change in SLD over time in individual patients are reported in ref. [47], where the effect of therapy on patients who received 10 mg of everolimus (patients A, B, C, D, E, F, G, H, I, J, K) has been observed. Some of them (A, B, C, D, E, F) received a placebo before the first drug treatment, so their observed tumor growth reflects the evolution without therapy. Other patients (G, H, I, J, K) received 10 mg from the beginning.

Consequently, the analysis of the two patient subgroups differs. For the A–F group, tumor growth without treatment is assessed, allowing the cumulative effect of therapy to be quantified. For the G–K group, the analysis aims to determine whether extrapolating from the limited initial subset of data can accurately describe tumor progression over a longer period.

It is important to note that when the number of data points is insufficient, it becomes difficult to distinguish between an exponential trend and the GL. Therefore, when necessary, a comparison will be made between the exponential trend (which corresponds to determining the product $k\ln(V_{\infty }/V_{0})$ for kΔt≪1) and the GL behavior with a fixed value of *k*, experimentally determined in vivo (as discussed below). This comparison is valuable because it highlights the effect when kΔt is not very small. Furthermore, the GL model of tumor evolution reveals a connection between the parameter *k* and the carrying capacity [42,45].

For the data from patients G, H, I, J, and K reported in Ref. [47], the effective GL parameters were fitted using the first three or four measurements. The SLD was evaluated and compared with the measured value after a long period (assuming spherical symmetry). To clarify this aspect, Table 3 reports the χ-square value for degree of freedom (d.o.f.), which shows very good agreement, with the exception of patient I. Indeed, Table 4 presents the percent residuals between the prediction (obtained using different sets of parameters) and the last observed data.

For example, for patient G, the prediction based on parameters fixed by the first four data points agrees with the last data point (50 days after the final input of the fitting procedure, i.e., the fourth data point) within 4.2 per thousand. The agreement with the extrapolation using the first three data points is within 1 percent, with a time difference of about 110 days. For patient J, the same analysis yields an agreement within 1.5 percent after 60 days and 1.6 percent after 180 days. In the worst case (patient I), the disagreement between data and extrapolation (using three data points to determine the GL parameters) is 13.8 percent after 120 days.

**Figure 6:**
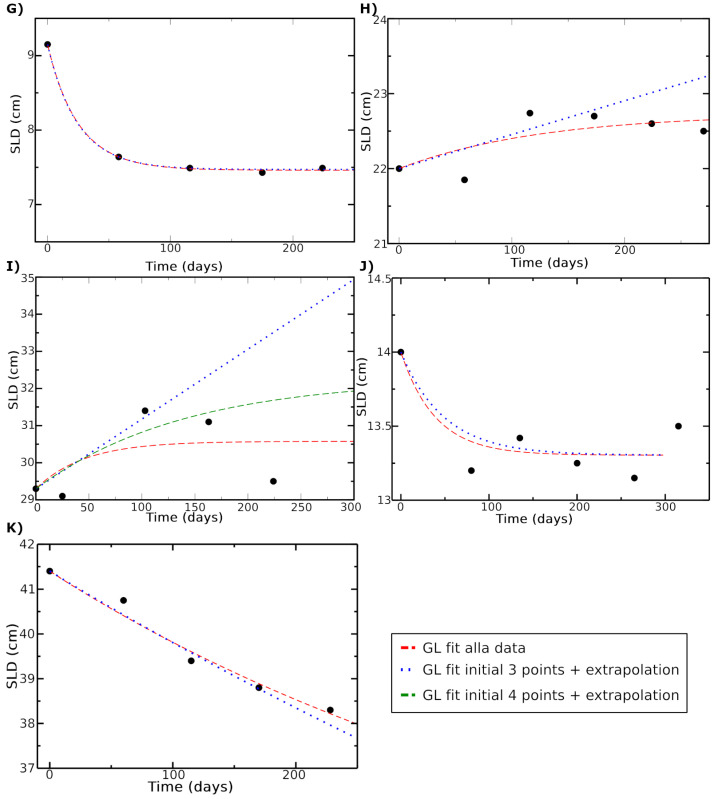
The predicted SLD values are compared with the measured values for patients G, H, I, J, and K. The GL fit for all data is represented by the red curve, while the GL fit using the initial three data points is shown by the blue dotted line. For patient I, the green curve represents the fit based on the first four data points. Data source [47].

This disagreement arises, as discussed earlier, from the small number of initial data points (three points) used for fitting. In such cases, it becomes impossible to differentiate the value of the carrying capacity from the value of *k*: only their product can be determined, which leads to an exponential behavior. However, the actual curve follows a Gompertz pattern, with the usual saturation at later times. Indeed, the GL fit using four initial data points is shown (green line) for patient I (Figure 6).

Therefore, the serial measurements in ref. [47] provide valuable examples of how initial monitoring can inform clinical decision-making.

For patients A, B, C, D, E, F, reported in ref. [47], there is an initial placebo administration, corresponding to the untreated evolution, followed by the therapy with everolimus. According to the general method in the previous subsection, data from the placebo period are used to determine the GL parameters for untreated tumor growth, while subsequent observations (starting from the first dose) provide a quantitative evaluation of the therapy effects.

As will be shown, this is indeed the final outcome, with one caveat: reliable determination of the untreated GL parameters is challenging due to the limited data during the placebo period. Therefore, to illustrate the computational approach, we constrain the parameter *k* to its average value in vivo [55]. While this partially compromises the patient-specific approach, it preserves the property of tumor size saturation by fitting the carrying capacity for every patient. This procedure is consistent with the observed correlation between GL parameters [42,45]. In fact, a small data set can be easily fitted using a single-parameter exponential pattern. However, extrapolating this to later stages (during treatment) could lead to an inaccurate assessment of the therapy effects and the missing threshold therapy for complete or partial recovery. Without accounting for saturation, the untreated tumor growth is unrealistically modeled as unbounded and exponential tumor growth can occur only initially. Subsequently, due to the limitation of nutrient supply (glucose, oxygen, etc.), mitosis must slow down and balance cell deaths, reaching a saturation phase. Nonetheless, evaluating the difference between an untreated exponential pattern and the GL for the patients under consideration can be useful for a quantitative understanding of this aspect.

**Figure 7:**
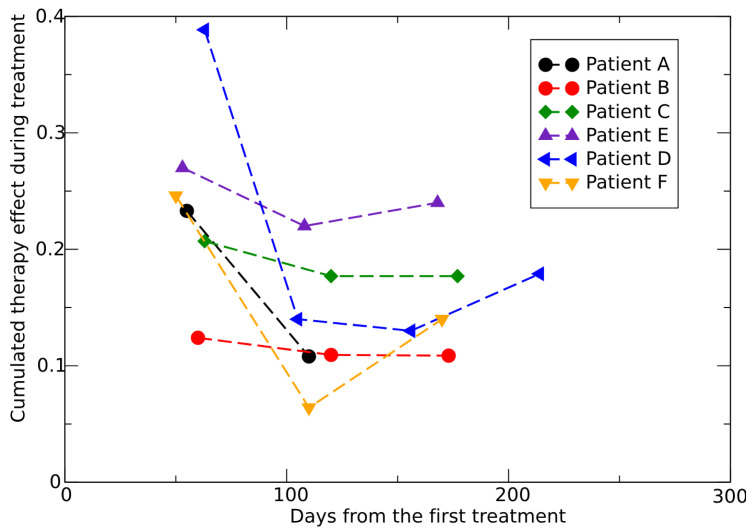
Cumulative effect of the therapy during treatments see Equation (6), fitted by data in ref. [47]. The parameter *k* in the GL is fixed at the average value 0.0342 day^−1^ [55]. The first point of each curve refers to the time interval after the first dose, the second point corresponds to the time interval between the first and the second dose and so on.

**Figure 8:**
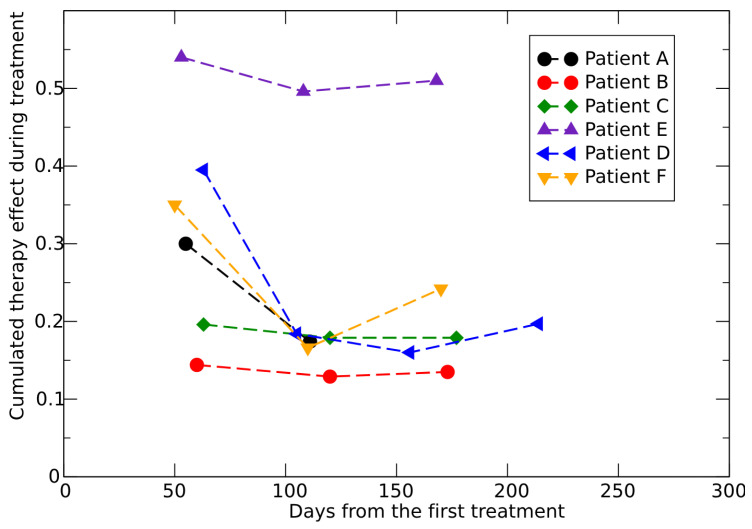
Cumulative effect of the therapy during treatments, fitted by data in ref. [47]. The untreated growth follows an exponential pattern. The first point of each curve refers to the time interval after the first dose, the second point corresponds to the time interval between the first and the second dose and so on.

The placebo data to determine the CC for untreated growth and the SLD evolution for six patients (patients A, B, C, D, E, F, see ref. [47]) provides a quantitative assessment of the therapy effects, as shown in Figure 7. The same results but assuming an untreated exponential growth are reported in Figure 8.

A clearer understanding of the results is provided in Table 5 which contains, for the specific patients, the ratio between the size before the second dose, $V(2^{-})$, and the size before the first one, $V(1^{-})$, then the ration between $V(3^{-})$ and $V(2^{-})$, and so on. The other columns report the different contribution (in %) to the size reduction due to the combination of regrowth, RG, and therapy, TH, (see Equations (9) and (10)). For example, for patient A, the volume reduction V2/V1 of 14% (1.0–0.86) is due to a regrowth of 16% (1.16) and a 26% cumulative therapy cell killing effect (1–0.74).

The GL fits of the placebo evolution give a patient dependent threshold for the critical value of the therapy effect for complete recovery: the cumulative effect of the therapy has to be TH<0.84,0.88,0.812,0.8,0.56,0.79 for patient A, B, C, D, E and F, respectively (see Equation (10)).

The previous analysis refers to patients receiving a specific dose, *d*, of 10 mg of everolimus. However, the effect of the therapy is dose-dependent, meaning that the value of TH is a function of *d*. Although determining this dependence requires a microscopic model, an immediate estimate of the critical dose, *d*, that induces tumor regression can be obtained by comparing the data on responses to different doses of pharmacological treatment.

In ref. [47], for another group of patients, the drug dosage was adjusted during the treatment period, decreasing from 10 mg to 5 mg of everolimus. This change in dosage resulted in a modification of the temporal evolution of tumor size. The determination of the patient-adapted critical dose can be derived from the analysis of this variation. Due to the very small amount of available data with different doses, we report, as an illustrative example, the temporal progression for patient P [47], which shows an initial reduction of the SLD with a dose of 10 mg, followed by regrowth due to the reduction to 5 mg. The experimental points allow an approximate estimate of the critical dose by comparing the parameter variations in the two phases. Whether considering exponential trends or the GL model, the slope of the curves changes sign when transitioning from a 10 mg dose to a 5 mg dose. Specifically, the exponential fits give a slope −0.0011 day^−1^ during the 10 mg tratment phase and 0.00135 day^−1^ for 5 mg. A simple linear interpolation indicates the critical dose of 7.8 mg for size reduction.

In conclusion, these examples show that monitoring the tumor evolution during therapy, following the suggested procedure, gives clinical, patient-oriented indications on: (1) the untreated growth; (2) the cumulative therapy effect for complete recovery; (3) an evaluation of the reduction for late time; and (4) the critical dose to obtain the size reduction.

### 3.5. Above Critical Dose

If therapy exceeds the critical dose at the beginning, tumor volume decreases after initial doses and continues to shrink during follow-up. In this scenario, tumor evolution can be assessed by fitting the monitored tumor size data directly using the GL curve with effective parameters. The evolution after the end of therapy is given by (we assume $k_{eff}>0$)

(26) $$V\left(t\right)/V\left(t_{e}\right)=e^{\left[ln\left(V_{eff}^{\infty }/V\left(t_{e}\right)\right)\right]\left[1-exp\left(-k_{eff}\left(t-t_{e}\right)\right)\right]},$$

where $t_{e}$ and $V(t_{e})$ are the time and the tumor volume at the end of therapy. Therefore, the fitted value of the CC, $V_{eff}^{\infty }$, gives the following indications:

- A very small CC compared to the final observed tumor volume, $V(t_{e})$, indicates a complete response;
- A CC close to $V(t_{e})$ gives an almost equilibrium condition, with a slow evolution after the end of therapy, that is generally classified as PR;
- For a CC much larger than $V(t_{e})$, the tumor can rapidly regrow.

As shown in the previous section, it is possible to predict the effects of therapy with a limited number of tumor-size observations. In this case, $t_{e}$ and $V(t_{e})$ refer to the last data point used in the fitting procedure. This aspect is particularly important when it is not clinically feasible to obtain an adequate time series of measurements. For clinical decisions that require a long interval of time after the end of therapy (e.g., surgery for colorectal cancer), a limited time series of observations during therapy should be reliable but a measurement of the tumor size evolution immediately after the end of therapy is crucial to understand the continuity of the effect once the patient is no longer receiving the treatment.

Let us now consider an example of clinical decision support based on Figure 5, assuming we only know the data on tumor size reduction during therapy, which allows an assessment of the effective GL parameters. Using these parameters, we can extrapolate tumor progression after the end of therapy (green curve in Figure 5). It is observed that the forecast indicates a slight reduction in size and that this reduction is reaching a saturation point (decreasing from about 35% to 27% over approximately 36 days). Based on this, the clinician can decide to initiate the intervention, thus avoiding possible complications due to delay. Conversely, if the forecast had indicated a sharp decrease in size, a longer wait would have facilitated the surgical intervention.

It is important to note that the proposed approach is patient-oriented, relying on individualized monitoring of disease evolution. A dose that is critical for one patient may be subcritical and produce slow or minimal effects in another.

### 3.6. Below Critical Dose

If, during monitoring, a growth pattern is observed, though slower than in the no-treatment scenario, the therapy effect is subcritical. In this case, since the GL parameters for the no-treatment condition have already been assessed, it is possible to estimate the critical dose, as suggested in Section 1.

For example, let us assume one has monitored the values RT(1),RT(2),RT(3). Based on these data, one can extrapolate the trend over time. This information is crucial for determining whether the fourth dose initiates the evolution towards complete response, that is if

(27) $$\left[ln\frac{V_{\infty }}{V(1^{-})}\right]\left[1-e^{-3k\Delta t}\right]<RT\left(1\right)e^{-3k\Delta t}+RT\left(2\right)e^{-2k\Delta t}+RT\left(3\right)$$

Tumor volume measurements $V\left(0\right),V\left(\tau_{1}\right),V\left(t_{1}^{-}\right),V\left(t_{2}^{-}\right),V\left(t_{3}^{-}\right)$ are necessary for performing the predictions.

It is important to emphasize that, after collecting the no-treatment data ($t=0,t=\tau_{1},t=t_{1}^{-}$), a continuous time series of observations (for doses 2, 3, 4, …) is not required. Instead, measurements can be taken at substantially different time intervals.

## 4. Discussion

The key challenge in mathematical modeling lies in achieving clinical translatability with a patient-centered focus. Bridging the gap between basic science and practical applications that can significantly improve patient outcomes is the most crucial objective.

Microbiological models are valuable for enhancing our dynamic understanding with a broader set of parameters. However, these models often exceed the practical clinical applicability threshold, which significantly limits their chances of being adopted in clinical settings [20,21,22,23].

Indeed, they rarely contain fewer than ten parameters and some of these are impractical or impossible to measure clinically, often requiring procedures that patients are unlikely to tolerate (such as multiple biopsies). This creates a disconnect between mathematical and clinical approaches, with limited opportunities for feasible clinical integration (see ref. [23]).

Studies modeling the effects of FLASH therapy offer a paradigmatic example. While they can capture important details, they do so at the cost of requiring numerous parameters and initial conditions that are not patient-oriented (see for example ref. [56]).

Our method advances toward clinical applicability by using practical and feasible tools with minimal impact on patient health (see [20] for a similar approach in hematology).

Monitoring tumor evolution during therapy is valuable to support personalized clinical decisions and requires a time series of tumor volume measurements to accurately predict long-term outcomes. The best clinical method is MRI and, in this respect, our quantitative analysis is complementary to other MRI-guided adaptive radiotherapy [8]. As discussed, by monitoring changes in tumor volume and in other biological characteristics, the treatment plan can be adjusted based on the observed treatment response and its quantitative analysis.

Our results and the examples in Section 3 also highlight the limitations of the proposed method. It can be applied to evaluate the effects of therapy when there are sufficient data on growth without treatment. Clinically, this is difficult to obtain, although a few data points on tumor size evolution after the first observation (at least three data points) would be sufficient to determine the untreated growth parameters. In the other case of tumor regression after the first therapeutic dose, the method requires initial monitoring only, which allows for the prediction of the progression evolution.

In the proposed computational method, the therapy effects have been described independently of the specific underlying biological model, by the introduction of GL effective parameters, *k* and the carrying capacity (see for example [33,38]).

In particular, in ref. [57], the method of effective carrying capacity was applied to assess the effects of radiotherapy on patients with head and neck tumors, assuming logistic growth for untreated tumors. The modification of the carrying capacity due to radiation is considered instantaneous after each treatment. More precisely, the computational strategy entails parameter tuning using the first cohort data of 17 patients; the validation of the tuned parameters on a different cohort of 36 patients and, then, in predicting the patient outcomes (defined as time without recurrence or cancer in the treated fields and disease-free survival) and risk assessment through a leave-one-out analysis using the combined data from both cohorts.

In our approach, the effective GL parameters are derived from observations made during the initial phase of treatment, capturing the cumulative effects of therapy. This allows for personalized prediction of long-term tumor evolution after therapy completion, without making assumptions about instantaneous or delayed treatment effects.

The new macroscopic parameterization of FLASH therapy effects is, in our opinion, particularly useful, as this technology is still largely immature and there is currently very little clinical evidence supporting these benefits [58,59]. However, FLASH therapy promises to revolutionize cancer treatment by the potential ability to safely test very high radiation doses never used before in clinical practice because of the well-known tolerance concerns. Due to the lack of sufficient knowledge of all the biological effects of this new therapy, a one-size-fits-all FLASH dose, capable of eradicating all cancer cells in every patient, is utopian, but it cannot be excluded. The road to such a goal is very long and full of obstacles. Meanwhile, the determination of cancer- and patient-specific critical doses can provide useful data for next-future tests. In this context, a possible correlation between parameters and biodynamics is discussed in the model presented in ref. [60].

## 5. Conclusions

The advantages and limitations of the proposed computational method are essentially linked to the following points:

(1) It is based on the patient-specific initial response to therapy;
(2) Only two effective parameters are needed to characterize this response, depending on whether the dose is below the critical threshold (i.e., whether there is immediate tumor shrinkage);
(3) It does not require specialized software, as any standard minimization routine can be used;
(4) It is fully general and can be applied to any tumor phenotype.

Data availability, however, is limited because monitoring during therapy is typically not performed in radiotherapy, or in chemotherapy it is performed only after a long interval.

A clear example is colorectal cancer, where monitoring of neoadjuvant radiochemotherapy usually takes place only at the end of treatment. From clinical point of view, this is completely understandable. On the other hand, the proposed approach requires, for example, two to three magnetic resonance imaging scans over approximately 3–4 weeks during neoadjuvant treatment with the advantage of the ability to predict long-term therapy effects, which, when combined with standard criteria, such as RECIST and biomarkers, can support clinical decisions about the timing of surgery or potential treatment modifications.

## Figures and Tables

**Table 1:** GL effective parameters for different cell lines and doses *d* in Gy. $d_{c}$ is the evaluated critical dose. The cell line 229 does not show any critical behavior (-); n.a. = not available. Data from ref. [39]. For cell lines 136,196,229 the parameter is in day^−1^.

Cell Line	Parameter	d = 0	d = 5	d = 8	d = 10	c	λ	$d_{c}$
36	$V_{d}^{\infty }/V\left(0\right)$	2.1	1.82	0.53	n.a.	0.00063	3.76	7.1
136	$ln\left(V_{d}^{\infty }/V\left(0\right)\right)k_{d}$	0.01853	n.a.	0.0175	−0.0206	3.85 × 10^−17^	15	9.5
196	$ln\left(V_{d}^{\infty }/V\left(0\right)\right)k_{d}$	0.0783	−0.0095	n.a.	−0.043	0.038	0.5	4.2
229	$ln\left(V_{d}^{\infty }/V\left(0\right)\right)k_{d}$	0.0234	0.019	n.a.	0.016	-	-	-

**Table 2:** Parameters of the FLASH therapy parameterization for different doses. $c_{0},c_{1},c_{2}$ are in day^−1^, $c_{f}$ is in day^−2^.

Parameter	15 Gy FLASH	19.5 Gy Conv.	20 Gy FLASH	25 Gy FLASH
$c_{0}$	0.067 ± 0.005	0.065 ± 0.004	0.0834 ± 0.004	0.056 ± 0.004
$c_{1}$	0.127 ± 0.043	0.084 ± 0.017	0.304 ± 0.13	0.166 ± 0.018
$c_{2}$	0.32 ± 0.1	0.073 ± 0.016	1.086 ± 0.26	0.159 ± 0.002
$c_{f}$	0.00052 ± 0.0001	0.00029 ± 0.00009	0.00044 ± 0.00005	−0.00043 ± 0.00013

**Table 3:** χ-square for d.o.f for the fit of all data conducted with the parameters fixed by the complete data set (column 2), by the first four and three data points (columns 3 and 4, respectively)—2.5% measurement error.

Patient	Parameters by All Data	Parameters by 4 Data	Parameters by 3 Data
G	0.17	0.18	0.27
H	0.25	0.55	0.78
I	1.28	2.56	8.57
J	0.2	0.2	0.21
K	0.043	0.051	0.056

**Table 4:** Residue in % between the last data point and the predicted value, when the GL parameters are determined by the whole data, by four and three data points, respectively. The days indicate the final day of observation after the initial observation (first column), and the time delay (days) after the last used data input to fit the parameters.

Patient	Parameters by All Data	Parameters by 4 Data	Parameters by 3 Data
G	0.31%—224 days	0.42%—50 days	1.02%—108 days
H	0.66%—270 days	2.67%—46 days	3.35%—154 days
I	3.5%—260 days	7.4%—60 days	13.8%—120 days
J	1.45%—315 days	1.55%—60 days	1.6%—180 days
K	0.22%—230 days	0.62%—60 days	0.74%—115 days

**Table 5:** Reduction factor due to the therapy (TH) and regrowth (RG) during therapy for specific patients (A, B, C, D, E, F). Vn is the value before dose *n*. The untreated growth follows the GL.

Patients	V2/V1	V3/V2	V4/V3	V5/V4
A	0.86	0.98	-	-
$RG_{A}$	1.16	1.16	-	-
$TH_{A}$	0.74	0.84	-	-
B	0.97	0.98	0.97	-
$RG_{B}$	1.12	1.12	1.11	-
$TH_{B}$	0.86	0.88	0.87	-
C	0.98	≃1	≃1	-
$RG_{C}$	1.2	1.2	1.2	-
$TH_{C}$	0.82	0.83	0.835	-
D	0.82	0.99	1.03	≃1
$RG_{D}$	1.22	1.19	1.21	1.22
$TH_{D}$	0.67	0.83	0.85	0.82
E	0.94	≃1	≃1	-
$RG_{E}$	1.63	1.64	1.66	-
$TH_{E}$	0.58	0.61	0.6	-
F	0.85	1.039	0.96	-
$RG_{F}$	1.2	1.22	1.23	-
$TH_{F}$	0.7	0.85	0.78	-

## Data Availability

All data generated or analyzed during this study are available on request to the corresponding author and included in the published articles and their Supplementary Information Files. In particular, data from ref. [39] are available on request; for data on FLASH therapy, see [46]; for data on neoadjuvant therapy in colorectal cancer, see [54]; for data on dose–response in metastatic renal cell carcinoma, see [47].

## Supplementary Materials

The following supporting information can be downloaded at: https://www.mdpi.com/article/10.3390/jpm15070275/s1.
