# Supplementary material for: Quantitative Method for Monitoring Tumor Evolution During and After Therapy
Source: J Pers Med. 2025 Jun 28;15(7):275. doi: 10.3390/jpm15070275 (PMC12299402; doi:10.3390/jpm15070275)
Supplement: Supplementary file 1 [file jpm-15-00275-s001.zip › jpm-3661838-supplementary.pdf]

1 S1

1.1 Effective parameters and therapy effects

An untreated tumor grows according to the Gompertz law (GL), solution of the equation

$$\frac{1}{V}\frac{dV}{dt} = a - kln(\frac{V(t)}{V(t_0)}) = kln(\frac{V_\infty}{V}) \quad \text{(S1)}$$

with two parameters, a, k or k, V_∞, where the carrying capacity, V_∞, is given by $V_\infty/V(t_0) = exp(a/k)$.

Let us consider the role of the therapy $F(t)$, i.e. let us modify the specific growth rate (the second term in eq.(1)) according to

$$\frac{1}{V}\frac{dV}{dt} = a - kln(\frac{V(t)}{V(t_0)}) - F(t) = kln(\frac{V_\infty}{V}) - F(t) \quad \text{(S2)}$$

where the variable $F(t)$ generically refers to the effect of any therapy (radio, immune,chemo) and/or to their combination.

The solution of eq.(28) is

$$\begin{aligned} V(t) &= V(t_0)e^{(a/k)[1-e^{-k(t-t_0)}] - \int_{t_0}^{t} dt^{'} F(t^{'})e^{-k(t-t^{'})}} \\ &= V(t_0)e^{[ln\frac{V_\infty}{V(t_0)}][1-e^{-k(t-t_0)}] - \int_{t_0}^{t} dt^{'} F(t^{'})e^{-k(t-t^{'})}}. \end{aligned} \quad \text{(S3)}$$

The limit $t \to \infty$ clarifies the mathematical definitions of complete response (CR) and partial response (PR). Indeed one gets CR or PR if

$$ln\frac{V_\infty}{V(t_0)} - lim_{t\to\infty}\int_{t_0}^{t} dt^{'} F(t^{'})e^{-k(t-t^{'})} \quad \text{(S4)}$$

goes respectively to $-\infty$ or to a negative finite value. For example, if $F(t) \simeq t$ one obtains CR, whereas for constant $F(t) = c$ one gets PR if $ln/V_\infty/V(t_0) - c/k < 0$.

On the other hand, at the clinical level, there are many parameters to define CR or PR and there is no way to observe a complete tumor cell extinction. Therefore a more empirical difference between CR and PR consists in the observed very large depletion of the tumor size for CR with respect to a moderate shrinkage for PR (it is useful to recall that the metabolic activity can be detected, by PET for example, only for size of order of mm).

Let us now discuss in detail how the therapy effect can be reabsorbed by a redefinition of the GL parameters a, k or V_∞, k. Let us start with a first " brute force" approach by defining

$$V(t) = V(t_0)e^{[ln\frac{V_\infty^{eff}}{V(t_0)}][1-e^{-k_{eff}(t-t_0)}]}, \quad \text{(S5)}$$

and comparing with eq.(29). The possible solutions, with the corresponding limitations, are:

1)

$$V_\infty^{eff} = V_\infty e^{-\int_{t_0}^{t} dt^{'} F(t^{'})e^{-k(t-t^{'})}} \quad \text{(S6)}$$

and

$$k_{eff} = k + \frac{1}{t-t_0} ln[1 - \frac{1}{ln[V_\infty/V(t_0)]} \int_{t_0}^{t} dt^{'} Ft^{'}) e^{-k(t-t^{'})}] = k + \frac{1}{t-t_0} ln[1 - \frac{ln(V_\infty^{eff}/V_\infty)}{ln(V_\infty/V(t_0))}] \tag{S7}$$

with the condition $\frac{V_\infty^{eff}}{V(t_0)} > 1$.

2) constant carrying capacity, $V_\infty^{eff} = V_\infty$. In this case

$$k_{eff} = k - \frac{1}{t-t_0} ln[1 + \frac{1}{ln[V_\infty/V(t_0)]} \int_{t_0}^{t} dt^{'} Ft^{'}) e^{-k(t-t^{'})}]. \tag{S8}$$

3)

$$ln \frac{V_\infty^{eff}}{V(t_0)} = -ln \frac{V_\infty}{V(t_0)} exp(-k(t-t_0) \tag{S9}$$

and

$$k_{eff} = -k - \frac{1}{t-t_0} ln[1 + \frac{1}{ln[V_\infty^{eff}/V(t_0)]} \int_{t_0}^{t} dt^{'} Ft^{'}) e^{+k(t^{'}-t_0)}]. \tag{S10}$$

acceptable only if $k_{eff} > 0$.

Therefore solution (1) can be applied far from the critical point $\frac{V_\infty^{eff}}{V(t_0)} = 1$; the second solution implies no effect of the therapy on the carrying capacity; the third one is very unlikely. Moreover there are other three not acceptable solutions giving $k_{eff} < 0$ and the solution with $k_{eff} = k$ given by

$$ln[\frac{V_\infty^{eff}}{V(t_0)}] = ln[\frac{V_\infty}{V(t_0)}] - \frac{1}{[1-e^{-k(t-t_0)}]} \int_{t_0}^{t} dt^{'} Ft^{'}) e^{-k(t-t^{'})} \tag{S11}$$

It is clear from the previous equations that , in general, the effective parameters could depend on both time and therapy. However, to better understand their role in tumor evolution, it is preferable to study the Gompertz equation with time and therapy-dependent parameters, along with the approximations that result in the description by the effective parameters, taking into account the specific time intervals of dose administrations (see appendix S2).

1.2 Time dependent effective parameters

A priori, there is no reason to assume that the effects of the therapy are time-independent. Let us therefore consider the Gompertz equation with time-dependent parameters, i.e.

$$\frac{1}{V} \frac{dV}{dt} = a(t) - k(t) ln[\frac{V(t)}{V(t_0)}]. \tag{S12}$$

By defining $y = ln(V/V(t_0))$, one gets

$$\frac{dy}{dt} = a(t) - k(t) y. \tag{S13}$$

The solution of previous equations is

$$V(t) = V(t_0) e^{\int_{t_0}^{t} d\tau a(\tau) e^{-[G(t,t_0) - G(\tau,t_0)]}}, \tag{S14}$$

where

$$G(t,t_0)=\int_{t_0}^{t}dt^{'}k(t^{'}) \tag{S15}$$

Let us now assume the therapy starts at t_0 with different doses at $t_1, t_2, ...t_n$, at regular intervals $t_n - t_{n-1} = \Delta t$. After the first dose and before the second one, the tumor evolution is given by

$$V(t_1)=V(t_0)e^{\int_{t_0}^{t_1}d\tau a(\tau)e^{-[G(t_1,t_0)-G(\tau,t_0)]}}, \tag{S16}$$

where, by mean value theorem,

$$G(t_1,t_0)=\int_{t_0}^{t_1}dt^{'}k(t^{'})=\bar{k}(1)(t_1-t_0), \tag{S17}$$

$$G(\tau,t_0)=\int_{t_0}^{\tau}dt^{'}k(t^{'})=\bar{k}(1)(\tau-t_0) \tag{S18}$$

and the same value $\bar{k}(1)$ has been considered in both integrals since the time interval Δt is small with respect to the typical time scale of the **GL** evolution ($O(1/k)$) and $t_0 < \tau < t_1$. Therefore, one gets

$$V(t_1)=V(t_0)e^{\int_{t_0}^{t_1}d\tau a(\tau)e^{-\bar{k}(1)(t_1-\tau)}}. \tag{S19}$$

By setting $a(t) = a_0 + a_1(t)$, with a_0 = constant, one obtains

$$\int_{t_0}^{t_1}d\tau a(\tau)e^{-\bar{k}(1)(t_1-\tau)}=\frac{a_0}{\bar{k}(1)}(1-e^{-\bar{k}(1)(t_1-t_0)})+e^{-\bar{k}(1)(t_1-t_0)}\int_{t_0}^{t_1}d\tau a_1(\tau)e^{-\bar{k}(1)(t_0-\tau)} \tag{S20}$$

By comparing the previous equation with the **GL effective parameters formula**

$$V(t_1)=V(t_0)e^{\frac{a_{eff}}{k_{eff}}(1-e^{-\bar{k}_{eff}(t_1-t_0)})} \tag{S21}$$

one determines that k_{eff} corresponds to the mean value $\bar{k}(1)$ after the first dose and

$$a_{eff}=a_0+\bar{k}(1)\frac{e^{-\bar{k}(1)(t_1-t_0)}}{1-e^{-\bar{k}(1)(t_1-t_0)}}\int_{t_0}^{t_1}d\tau a_1(\tau)e^{-[\bar{k}(1)(t_1-\tau)]}. \tag{S22}$$

The iteration during the time interval $t_2 - t_1$ yields similar results with $\bar{k}(2)$ = mean value after two doses, and a_{eff} determined by the corresponding formula. In conclusion, there is a time series of effective parameters, related to the different doses administration at different times.

2 S2

2.1 Evaluation of the cumulative effect of the therapy

Let us consider a therapy administrated at regular intervals at times $t_1, t_2, ..t_n = n\Delta t$ and let us call $V(n^-), V(n^+)$ the tumor volume before and after the $n-$th treatment.

According to GL, $V(2^-)$ and $V(3^-)$ as a function of $V(1^-)$ are given by (see eqs.(14-17) main text)

$$V(2^-)=V(1^-)e^{[ln\frac{V_\infty}{V(1^-)}][1-e^{-k\Delta t}]-RT(1)}, \tag{S23}$$

$$V(3^-)=V(1^-)e^{[ln\frac{V_\infty}{V(1^-)}][1-e^{-2k\delta t}]-RT(1)e^{-k\Delta t}-RT(2)}. \tag{S24}$$

Let us evaluate $V(4^-)$ as a function of $V(1^-)$ starting from

$$V(4^-) = V(3^-)e^{[ln\frac{V_\infty}{V(3^-)}][1-e^{-k\Delta t}]-RT(3)}. \tag{S25}$$

By previous eq.(24), it turns out

$$V(4^-) = V(1^-)e^{[ln\frac{V_\infty}{V(1^-)}][1-e^{-3k\Delta t}]-RT(1)e^{-2k\Delta t}-RT(2)e^{-k\Delta t}-RT(3)}. \tag{S26}$$

By iteration, one gets eq.(22) in the main text and eq.(23) by imposing the condition $V(n^-) < V(1^-)$.

If, on the other hand, a monotonic reduction in size is imposed, from the general relation

$$V[n^-] = V[(n-1)^-]e^{ln(\frac{V_\infty}{V[(n-1)^-]})[1-e^{-k\Delta t)}]-RT(n-1)}, \tag{S27}$$

one gets

$$V[2^-]/V[1^-] = e^{ln(\frac{V_\infty}{V[1^-]})[1-e^{-k\Delta t)}]-RT(1)} < 1 \tag{S28}$$

$$V[3^-]/V[2^-] = e^{ln(\frac{V_\infty}{V[2^-]})[1-e^{-k\Delta t)}]-RT(2)} < 1 \tag{S29}$$

and so on.

By substitution of $V[2^-]$ from eq.(28) in eq.(29), it turns out the condition

$$ln(\frac{V_\infty}{V[1^-]})[1-e^{-k\Delta t)}]e^{-k\Delta t)} + RT(1)[1-e^{-k\Delta t)}] - RT(2) < 0 \tag{S30}$$

By similar calculations for $V[4^-] < V[3^-]$ one finds

$$ln(\frac{V_\infty}{V[1^-]})[1-e^{-k\Delta t)}]e^{-2k\Delta t)}+RT(1)[1-e^{-k\Delta t)}]e^{-k\Delta t)}+RT(2)1-e^{-k\Delta t)}]-RT(3) < 0. \tag{S31}$$

The general rule for $V[n^-] < V[(n-1)^-]$ is given by

$$ln(\frac{V_\infty}{V[1^-]})[1-e^{-k\Delta t)}]e^{-(n-2)k\Delta t)}+[1-e^{-k\Delta t)}]\Sigma_{j=1}^{n-2}RT(j)e^{[j-(n-2)]k\Delta t}-RT(n-1) < 0 \tag{S32}$$

2.2 Correspondence between effective GL parameters and Flash therapy parametrization

This appendix is devoted to exemplify the correspondence between the GL effective parameters and the parametrizazion of $F(t)$ in a particular case. It is an application of Appendices A and B.

According to parametrization of $F(t)$,

$$F(t) = c_0 + c_1 * e^{(-c_2 t)} - c_f t, \tag{S33}$$

one gets

$$V(t)/V(0) = exp[(ln\frac{V_\infty}{V(0)} - c_0/k - c_f/k^2)(1-e^{-kt}) - \frac{c_1}{k-c_2}(e^{-c_2 t} - e^{-kt}) + tc_f/k]. \tag{S34}$$

which gives the relation with the GL effective parameters,

$$V(t) = V(t_0)e^{[ln\frac{V_\infty^{eff}}{V(t_0)}][1-e^{-k_{eff}(t-t_0)}]}, \tag{S35}$$

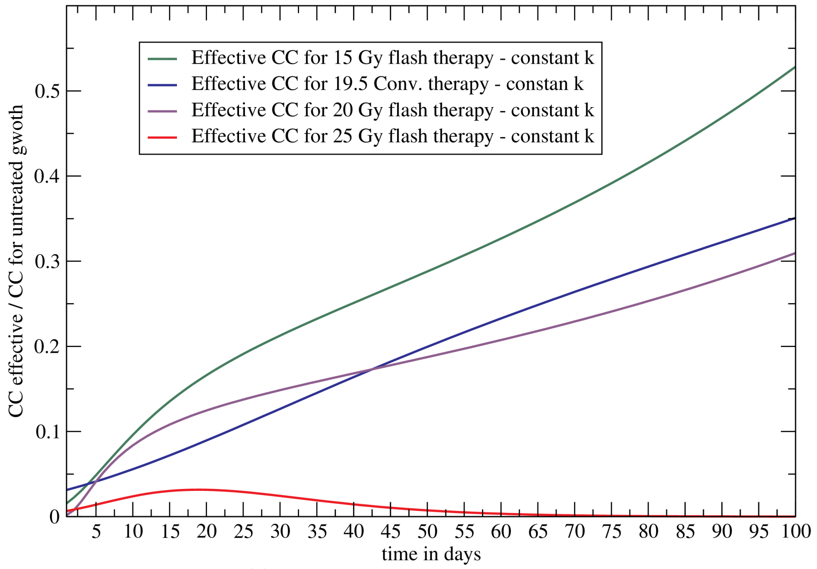

Fig 1. The ratio V_∞^{eff}/V_∞ as a function of time obtained by the $F(t)$ parameterization, assuming $k_{eff} = k$.

by equating the two previous equations. According to Appendices A and B, there are various solutions. For illustrative purposes only, let as assume that $k_{eff} = k$ and therefore one gets

$$V_\infty^{eff}/V_\infty = exp(-W) \tag{S36}$$

with

$$W = c_0/k + c_f/k^2 + \frac{c_1(e^{-c2t} - e^{-kt})}{(k - c_2)[1 - exp(-kt)]} - \frac{c_f t}{k[1 - exp(-kt)]} \tag{S37}$$

The result is depicted in Fig.1 and shows the time dependence of the effective carrying capacity with respect the untreated growth.
